# Safety and Efficacy of Ultrasound-Guided Combined Segmental Thoracic Spinal Epidural Anesthesia in Abdominal Surgeries and Laparoscopic Procedures: A Prospective Randomized Clinical Study

**DOI:** 10.5812/aapm-138825

**Published:** 2024-01-14

**Authors:** Alaa Ali M. Elzohry, Ahmed S. Hegab, Osama Yehia A. Khalifa, Khadeja M. Elhossieny, Fatma Al Zahraa H. Abdel Hameed

**Affiliations:** 1 Anasthesia, ICU and Pain Management, South Egypt Cancer Institute, Assiut University, El Fateh, Egypt; 2 Anasthesiology, ICU and Pain Management, Faculty of Medicine, Zagazig University, Zagazig, Egypt

**Keywords:** Combined Thoracic Spinal Epidural, General Anesthesia, Laparoscopic Surgeries, Acute Pain, VAS Scores

## Abstract

**Background:**

Thoracic segmental spinal anesthesia (SA) may be a good alternative to general anesthesia (GA) for abdominal operations and laparoscopic procedures, especially in high-risk patients.

**Objectives:**

The aim of this study was to investigate the safety and efficacy of thoracic segmental SA vs GA during abdominal operations and laparoscopic procedures.

**Methods:**

This study was conducted at our university hospital and involved a total of 46 patients who underwent abdominal operations and laparoscopic procedures. The study period spanned from January 15, 2022, to October 15, 2022. Patients were divided into 2 groups: Group 1 (n = 23) received standard GA, and group 2 (n = 23) received thoracic segmental SA. A combination of 10 mg of hyperbaric bupivacaine 0.5% and 25 μg of fentanyl was injected through the spinal needle. The epidural catheter was then threaded through the Tuohy needle after withdrawal of the spinal needle to keep only 4 cm up in the epidural space. Demographic data, both intra and postoperative hemodynamic parameters, were monitored. Postoperatively, pain in both groups was treated with intravenous (IV) morphine by patient controlled analgesia (PCA), PCA settings were 1 mg morphine/mL, no background infusion, bolus dose 2 mL and lockout interval 15 min. Postoperative, both resting VAS and VAS during cough were measured for all patients at fixed intervals, and all patients were followed up for postoperative complications.

**Results:**

No significant variation was found in demographic data. Intra and postoperative mean arterial pressure (MAP) and heart rate (HR) measurements were higher in group 1 than in group 2 but without a statistically significant difference (P < 0.029). Early postoperative VAS values and discharge time from the postanesthesia care unit (PACU) were significantly reduced in group 2 than in group 1 (P < 0.001). The number of patients asked for analgesia and total opioid consumption were substantially reduced in group 2 than in group 1. Also, the time of the first analgesia request and patient satisfaction were substantially greater in group 2 than in group 1.

**Conclusions:**

Combined thoracic spinal/epidural block results in stable hemodynamics, longer postoperative analgesia with fewer side effects, and greater surgeon and patient satisfaction in patients undergoing abdominal operations and laparoscopic procedures.

## 1. Background

Patients undergoing upper abdominal or thoracic surgery are substantially more likely to have postoperative respiratory failure than healthy patients if they have a prior pulmonary abnormality, such as severe chronic obstructive pulmonary disease (COPD) (1). Even in the overall population, people with comorbidities have a greater risk of problems from general anesthesia (GA) than those without (2). Numerous intra- and postoperative problems, including bronchospasm, laryngospasm, and extended mechanical breathing, may emerge from this. To lower the danger of severe problems, these patients must have the proper anesthetic procedure. Thoracic spinal anesthesia (SA) is one of the anesthetic methods that may be used for such individuals during operations like cholecystectomy (3).

Thoracic segmental SA is receiving increasing interest for several popular operations. It has been demonstrated that intrathecal injection of anesthetics into the optimal body height and above the spinal cord's terminus is advantageous in these particular circumstances (4).

Given these frequent procedures, thoracic SA may be an additional alternative with benefits to patient safety, recovery after anesthesia, and postoperative pain reduction (5).

Segmental thoracic SA with epidural anesthetic was selected because it eliminates the possibility of inadequate muscle relaxation for surgery and delivers high-quality analgesic, negating the need for large dosages of extra systemic analgesics (6).

With the advancement of the technology and its popularity, the ultrasound-guided approach has recently attracted attention (7).

However, using ultrasound guidance for nerve block has several significant practical benefits, such as identification of area of interest with clear anatomical landmarks (8). This enables safer navigation of the needle to the target while avoiding potential needle-damaging structures. Additionally, ultrasound makes it possible to see the needle tip as it is inserted into the tissues, ensuring that it is traveling along the desired course and once again lowering the risk of unintentional needle harm to nearby structures (9).

## 2. Objectives

The aim of this study was to investigate the safety and efficacy of thoracic segmental SA vs GA during abdominal operations and laparoscopic procedures.

## 3. Methods

This study was conducted at our university hospital from January 15, 2022, to October 15, 2022. The study included 46 subjects who underwent various surgical procedures, such as abdominal cholecystectomy, splenectomy, epigastric hernia repair, renal surgery, adrenal surgery, and laparoscopic surgery. The study was carried out after obtaining approval from the local ethical committee under code 9382 9/1/2022 (https://www.zu.edu.eg/). The review has a novel ID of NCT05587608 on ClinicalTrials.gov.

Inclusion criteria were (1) patients at risk, including older patients with declining physiological reserves, comorbidities, polypharmacy, cognitive disorders, and frailty; (2) patients aged 18 - 65 years; (3) patients scheduled for abdominal cholecystectomy; (4) patients who expressed unwillingness to undergo GA; (5) patients who were unable to undergo the standard SA technique in the lumbar area; and (6) patients who were unable to tolerate the traditional method of SA.

Exclusion criteria were preexisting neurological illnesses (multiple sclerosis and other demyelinating illnesses), sepsis, severe hypovolemia, and coagulopathy. Also, patients with local infection at the site of the operation, increased intracranial pressure, left ventricular outflow blockage, and significant mitral and aortic stenosis with symptoms of hypertrophic obstructive cardiomyopathy were excluded.

Demographic data regarding age, sex, type of operation, duration of operation, body mass index (BMI), and score of the American Society of Anesthesia (ASA) were recorded. At the time of examination, basic investigations were conducted, and the patient's written, fully informed consent was obtained before proceeding with any further steps or procedures. Prior to the induction of anesthesia, several monitoring devices were set up, including an electrocardiogram (ECG) machine, automatic non-invasive blood pressure measurement equipment, and a pulse oximeter.

Randomization in this study was conducted using a method involving serially numbered, opaque envelopes that concealed the randomization assignments generated by a computer-generated list. Patients were divided into 2 equal groups: Group 1 (n = 23) received standard GA, and group 2 (n = 23) received thoracic segmental SA. A combination of 10 mg of hyperbaric bupivacaine 0.5% and 25 μg of fentanyl was injected through the spinal needle. The epidural catheter was then threaded through the Tuohy needle after withdrawal of the spinal needle to keep only 4 cm up in the epidural space.

### 3.1. Technique of General Anesthesia

All patients in group 1 were ventilated with oxygen via a face mask, followed by laryngoscopy and tracheal intubation. The anesthesia was induced using 2.5 mg/kg of propofol, 2 μg/kg of fentanyl, 0.8 mg/kg of cisatracurium, and 1.5 mg/kg of lidocaine. Following intubation, the respiratory rate (RR) was changed to keep the end-tidal CO_2_ (EtCO_2_) between 33 and 36 mm Hg while maintaining a tidal volume of 8 mL/kg and positive end-expiratory pressure (PEEP) of 5 cm H_2_O. Sevoflurane (1% - 1.5%), muscle relaxant doses, and regulated mechanical breathing were used to accomplish maintenance. A gas analyzer was used to measure EtCO_2_ and sevoflurane continually. Following the surgery, 2 mg of neostigmine and 1 mg of atropine were administered to treat any remaining neuromuscular blockade.

Electrocardiogram, pulse rate, arterial blood pressure, respiration rate, pulse oximetry, and EtCO_2_ were continually measured during the procedure. All information was captured every 5 min.

### 3.2. Technique of Thoracic Segmental Spinal Epidural Anesthesia

**Figure 1.**
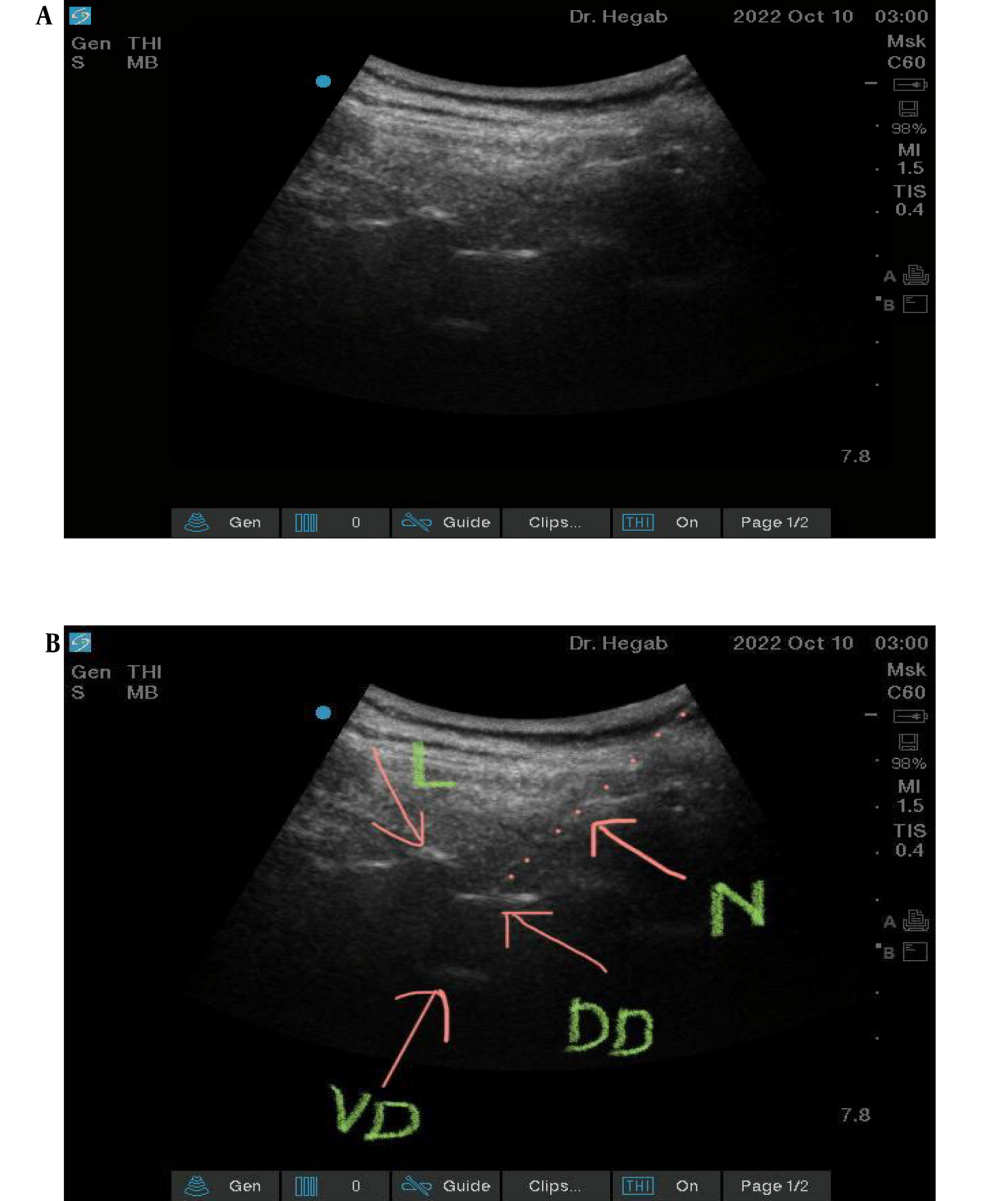
A and B, Technique of US guided segmental thoracic neuro-axial block (VD, ventral dura; DD, dorsal dura; L, lamina; N, needle).

Group 2 was given ultrasound-guided thoracic segmental SA at the level of T9 - 10 interlaminar space; patients were in a sitting position. We used a combined spinal epidural tray (PERIFIX B Braun, Melsungen, Hessen Germany) ^®^. The epidural needle was 17 Ga Touhy, while Pencil- the Point Spinal Needle was 27 Ga (Figure 1A and B).

In this group, we used a SonoSite C60x/5-2 MHz M-Turbo Convex Probe Ultrasound Transducer to identify the T10 interlaminar space, and after complete sterilization, we introduced the epidural needle in a paramedian in plan approach till the epidural space. The spinal needle was then introduced through the epidural needle not more than 0.5 cm beyond the dura mater. A combination of 2 mL of hyperbaric bupivacaine 0.5% and 25 ug of fentanyl was injected through the spinal needle. The epidural catheter was then threaded through the Tuohy needle after withdrawal of the spinal needle to keep only 4 cm up in the epidural space.

After testing the sensory level using a piece of ice and the motor level using a modified Bromage scale while the patient was in a supine position, surgery was started after achieving sufficient surgical anesthesia. At the same time, continuous infusion of isobaric bupivacaine in the epidural catheter started at a rate of 2 mL/h. During surgery, we gave drugs needed to treat anxiety, pain, hypotension, bradycardia, pruritis, nausea, and vomiting. Perioperative monitoring for hemodynamics, pain, anxiety, nausea, vomiting, and neurological symptoms were recorded, and all patients were followed up during their hospital stay and for 72 hours postoperative. If they were discharged, they would be followed up using a phone call.

Postoperatively, both groups: Pain was treated with IV morphine by patient controlled analgesia (PCA), PCA settings were; 1 mg morphine/mL, no background infusion, bolus dose 2 mL and lockout interval 15 min. Pain was monitored using the Visual Analog Scale (VAS) at rest and during cough every 4 h for 24 h. Hemodynamic parameters (including heart rate [HR], mean arterial pressure [MAP], and RR) were also monitored every 2 h for 24 h. Total opioid consumption by PCA was documented. Any negative side effects were noted, including bradycardia, hypotension, nausea, vomiting, urinary incontinence, and stomach pain. The total amount of opioids consumed and the first analgesic request were noticed and recorded. Patient satisfaction using Short Assessment of Patient Satisfaction (SAPS) and surgeon satisfaction based on a Likert scale were documented.

Each participant involved in the research study provided written informed consent, which was obtained after the project received approval from the university's ethics committee. According to the Declaration of Helsinki, each participant in the research provided written informed permission, which was obtained after the project was given the green light by the university's ethics committee.

The data were analyzed using SPSS version 24. The statistical significance was assessed using the analysis of variance (ANOVA) test, multiple linear regression, standard seaborn, Matplotlib boxplots, Wilcoxon's tests, and Spearman's correlation. Each variable was evaluated in accordance with the sort of data it held (parametric or not). P values less than 0.05 were deemed statistically significant findings.

## 4. Results

**Figure 2.**
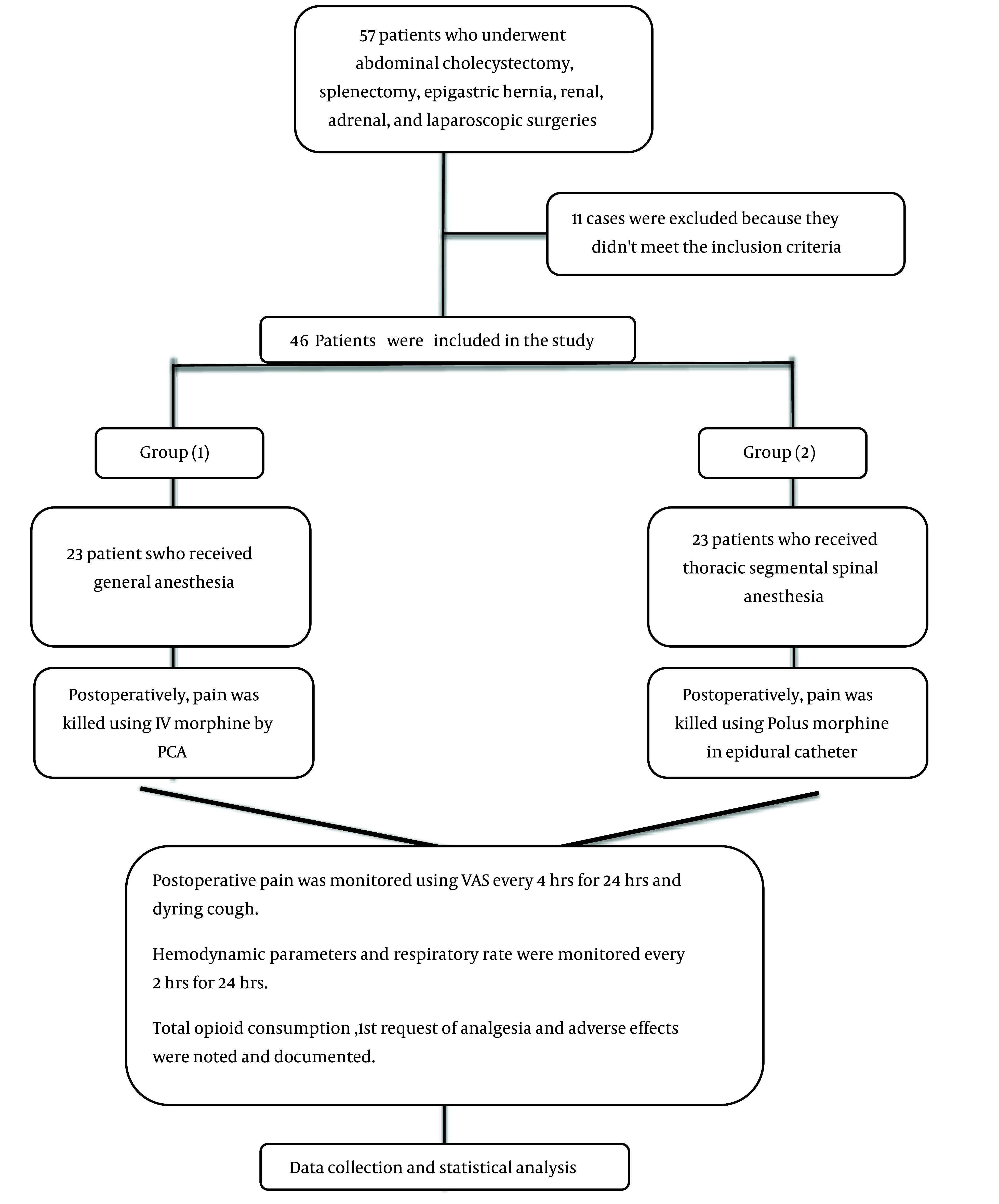
The flowchart of cases throughout the study (Abbreviations: PCA, patient-controlled anesthesia; VAS, Visual Analog Scale).

The flowchart of cases is illustrated in Figure 2. 

No considerable variance was found in basic data, as presented in Table 1. 

**Table 1.** Basic Data of the Patients Between the 2 Groups ^a^

Variables	Group 1 (N = 23)	Group 2 (N = 23)	P-Value
**Age (y), mean ± SD**	43.78 ± 8.47	42.68 ± 9.34	0.678
**BMI (kg/m** ^**2**^ **), mean ± SD**	26.72 ± 2.85	27.19 ± 3.28	0.607
**Sex**			0.760
Male	14 (60.9)	15 (65.2)	
Female	9 (39.1)	8 (34.8)	
**ASA classification**			0.835
ASA I	8 (34.8)	7 (30.4)	
ASA II	10 (43.5)	12 (52.2)	
ASA III	5 (21.7)	4 (17.4)	
**Comorbidities**			
Smoking	9 (39.1)	8 (34.8)	0.760
Hypertension	5 (21.7)	4 (17.4)	0.710
DM	3 (13)	4 (17.4)	0.681
**Operation type**			0.914
Cholecystectomy	7 (30.4)	6 (26.1)	
Splenectomy	3 (13)	4 (17.4)	
Laparoscopic	8 (34.8)	10 (43.5)	
Pancreatic surgeries	2 (8.7)	1 (4.3)	
Others	3 (13)	2 (8.7)	

Abbreviations: BMI, body mass index; ASA, American Society of Anesthesiologists; DM, diabetes mellitus.

^a^ Values are expressed as No. (%) unless otherwise indicated.

No significant difference was found in basic data, as presented in Table 1. 

**Figure 3.**
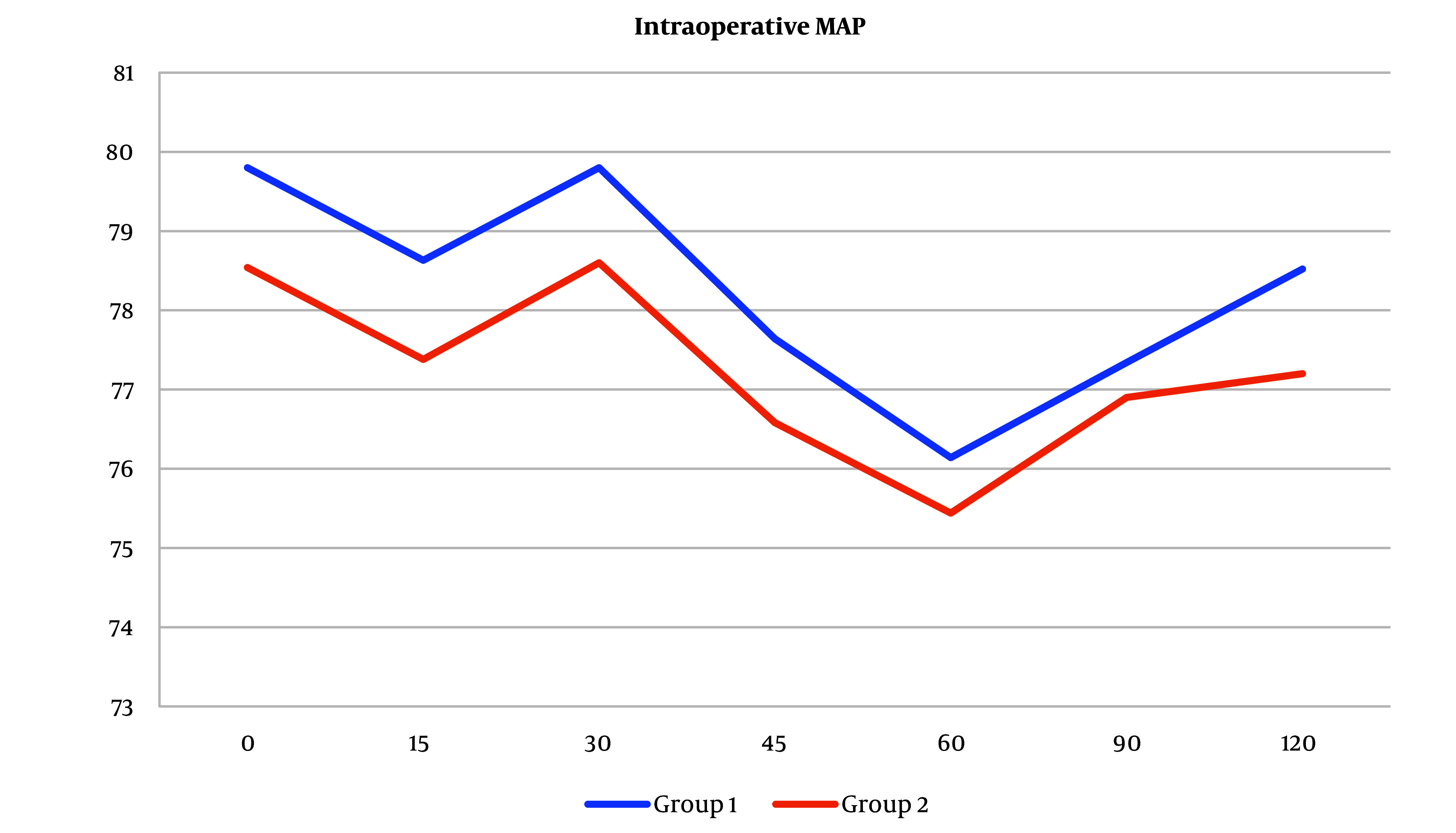
Mean arterial pressure (MAP) measurements intraoperatively between the two groups. Mean arterial pressure measures increased in group 1 than in group 2, but the difference was not statistically significant (Abbreviation: MAP, mean arterial pressure).

**Figure 4.**
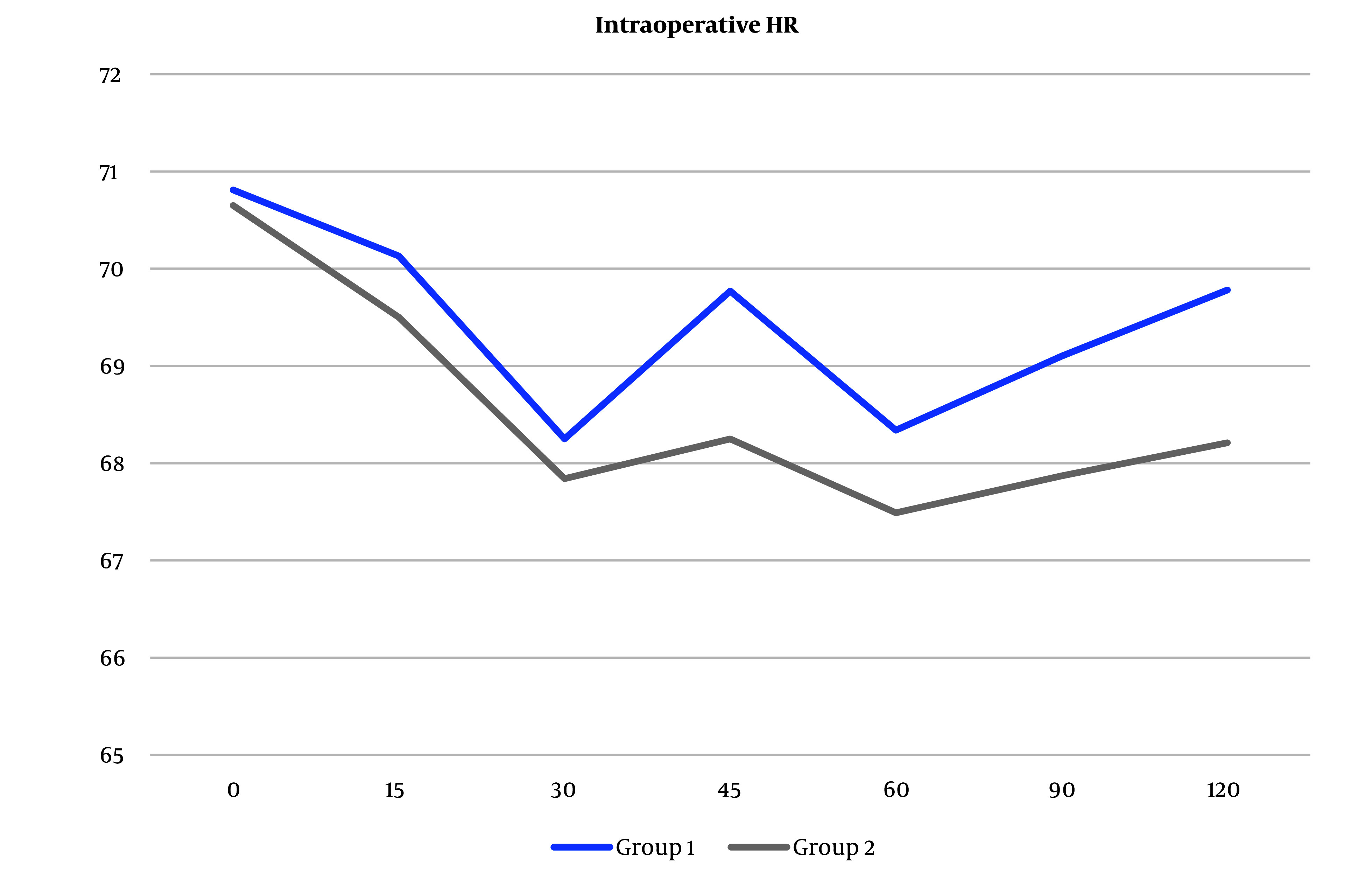
Mean arterial pressure measurements postoperatively between the 2 groups. Measures increased in group 1 than in group 2, but the difference was not statistically significant (Abbreviation: MAP, mean arterial pressure).

Intra- and postoperative MAP measures increased in group 1 than in group 2, but the difference was not statistically significant (Figures 3 and 4). 

**Figure 5.**
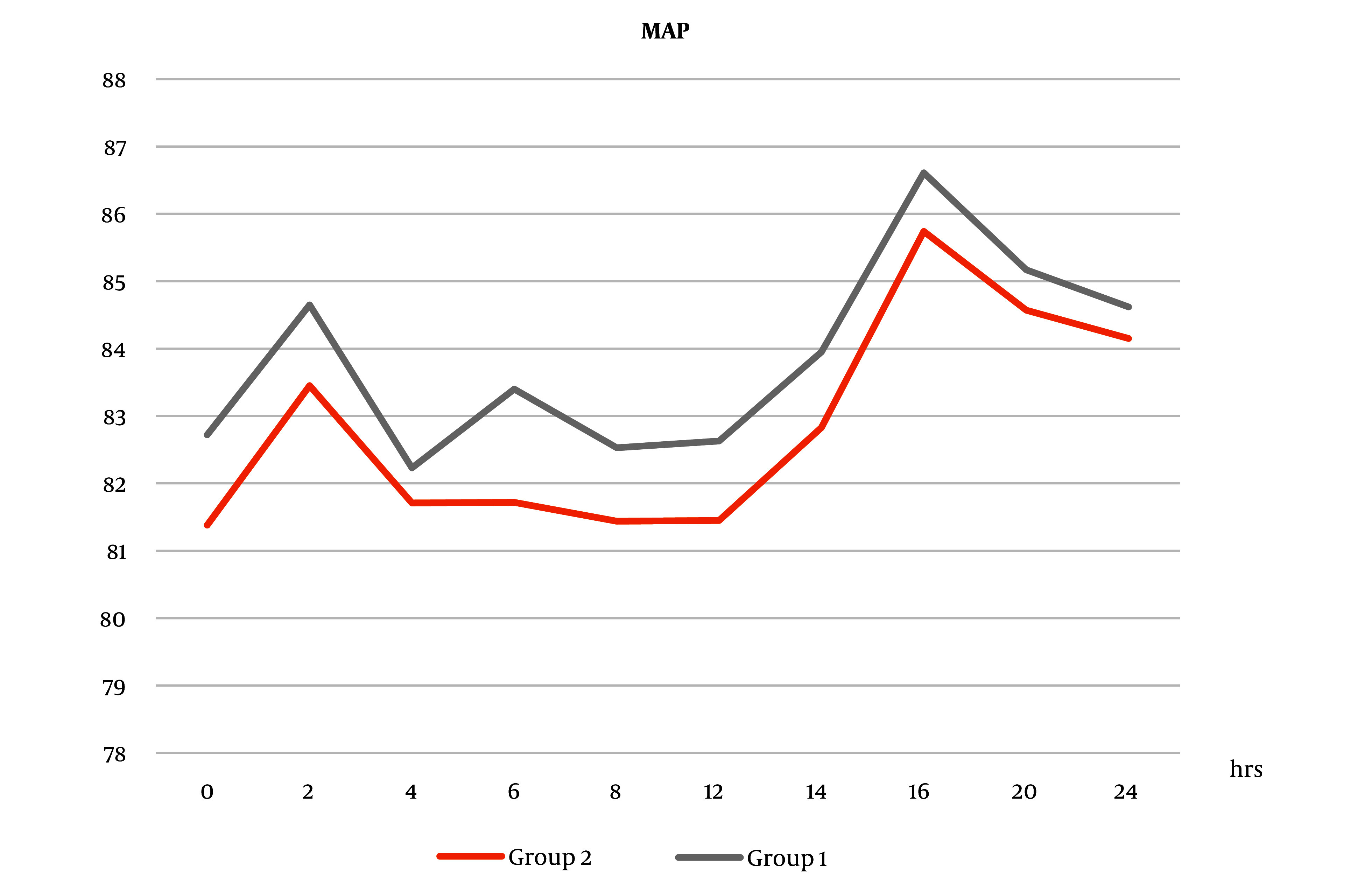
Heart rate (HR) measurements intraoperatively between the 2 groups. Heart rate measures increased in group 1 than in group 2, but the difference was not statistically significant (Abbreviation: HR, heart rate).

**Figure 6.**
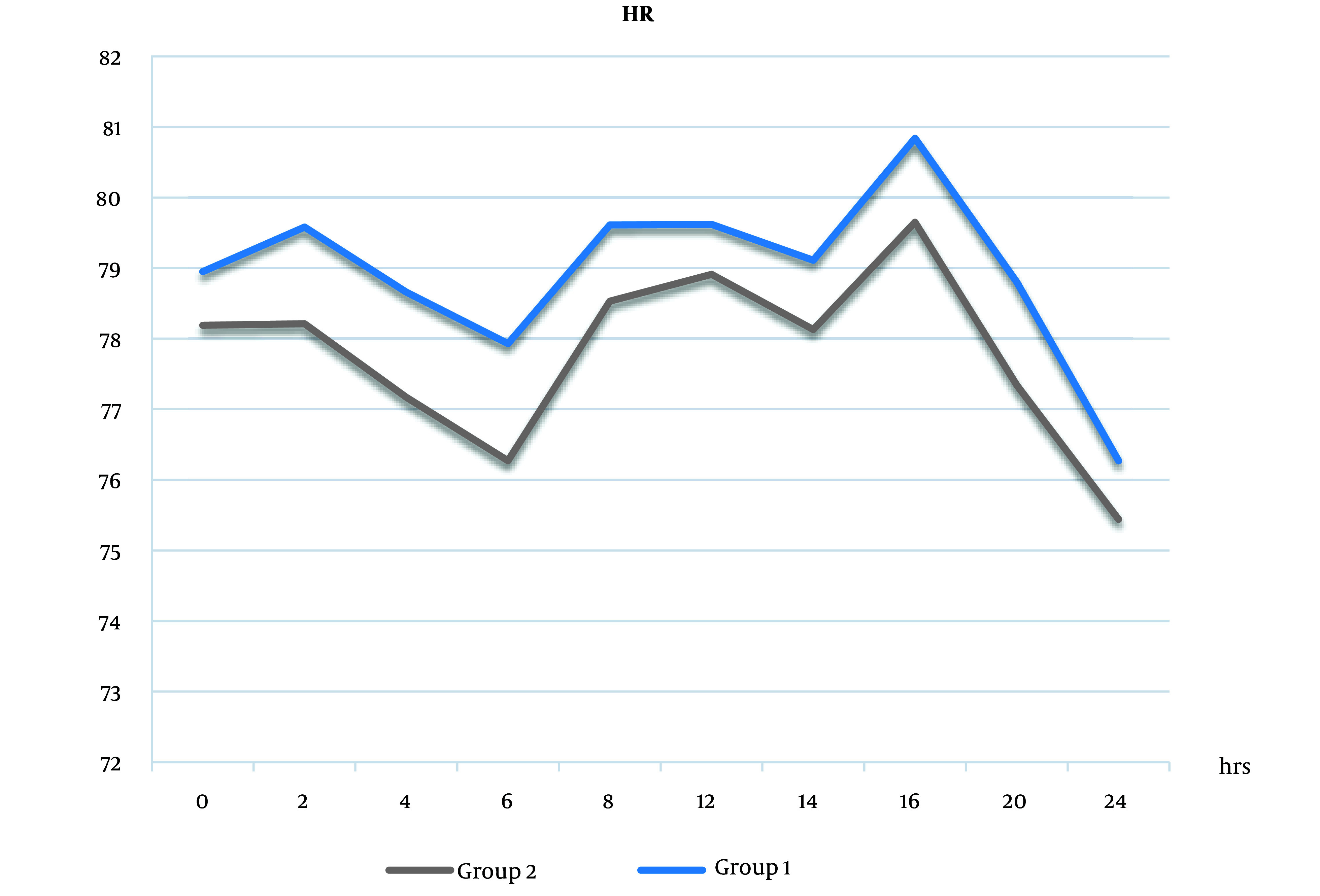
Heart rate measurements postoperatively between the 2 groups. Measures increased in group 1 than in group 2, but the difference was not statistically significant (Abbreviation: HR, heart rate).

The same was true in intra- and postoperative HR values (Figures 5 and 6). 

**Figure 7.**
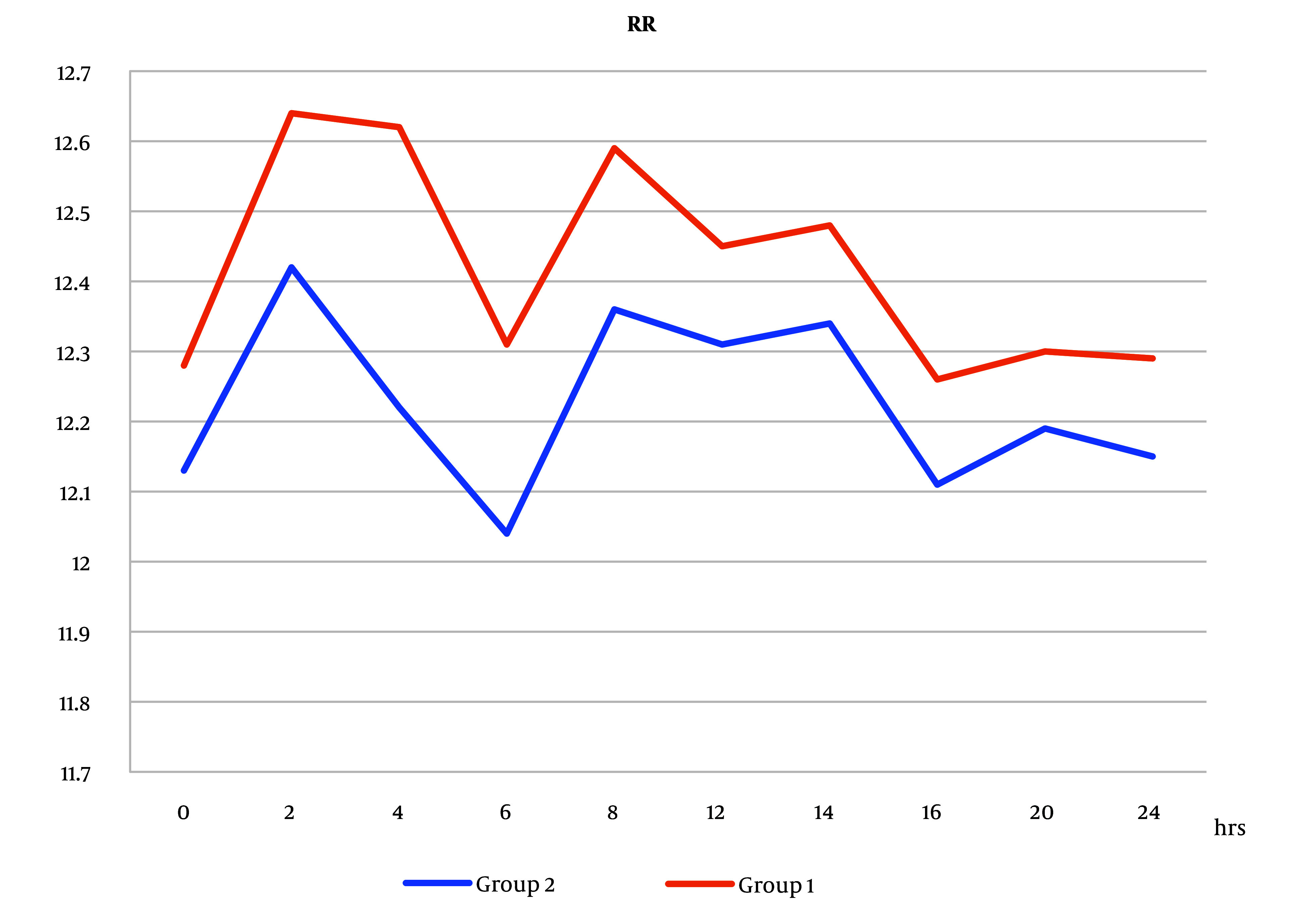
Respiratory rate (RR) measurements postoperatively between the 2 groups. Respiratory rate measures increased in group 1 than in group 2, but the difference was not statistically significant (Abbreviation: RR, respiratory rate).

However, there was no statistically significant difference in RR readings that increased in group 1 compared to group 2 (Figure 7). 

No significant difference was found between the groups regarding intraoperative data (Table 2). 

**Table 2.** Postoperative Data Between the 2 Groups ^a^

Operative Data	Group 1 (N = 23)	Group 2 (N = 23)	P-Value
**Operative time (min); mean ± SD**	121.83 ± 40.52	123.65 ± 41.28	0.881
**Intraoperative complications**			
Hypertension	0	1 (4.3)	0.918
Hypotension	2 (8.7)	0	0.148
Tachycardia	0	3 (13)	0.029
Bradycardia	3 (13)	0	0.073
Paresthesia	2 (8.7)	0	0.148
Pruritis	1 (4.3)	0	0.314

^a^ Values are expressed as No. (%) unless otherwise indicated.

No significant difference was found between the groups regarding intraoperative data.

The VAS values at rest and during cough significantly decreased in group 2 than in group 1 (Table 3). 

**Table 3.** Comparison of Postoperative Visual Analog Scale Pain Score Over the Study Time ^a^

VAS Scores (h)	Group 1 (N = 23)	Group 1 (N = 23)	P-Value
VAS 0 at rest	2.45 (2.00 - 3.70)	2.45 (2.00 - 3.00)	0.014 ^b^
VAS.4	2.80 (2.20 - 4.00)	2.60 (1.60 - 3.30)	< 0.001 ^b^
VAS.8	2.70 (2.20 - 3.20)	2.20 (1.70 - 3.40)	0.004 ^b^
VAS.12	2.75 (2.50 - 3.30)	2.40 (2.00 - 3.00)	0.001 ^b^
VAS.16	2.90 (3.00 - 3.50)	2.60 (2.50 - 3.00)	0.239
VAS.20	3.00 (3.00 - 3.70)	2.65 (2.60 - 3.10)	0.228
VAS.24	2.80 (2.70 - 3.00)	2.45 (2.40 - 2.90)	0.014
VAS.0 with cough	2.50 (2.50 - 3.00)	2.25 (2.25 - 2.70)	< 0.001 ^b^
VAS.4	2.90 (2.20 - 4.00)	2.80 (1.60 - 3.30)	0.004 ^b^
VAS.8	2.90 (2.20 - 3.20)	2.30 (1.70 - 3.40)	0.001 ^b^
VAS.12	2.95 (2.50 - 3.30)	2.50 (2.00 - 3.00)	< 0.001 ^b^
VAS.16	3.10 (3.00 - 3.50)	2.80 (2.50 - 3.00)	< 0.001 ^b^
VAS.20	3.40 (3.00 - 3.70)	2.75 (2.60 - 3.10)	0.024 ^b^
VAS.24	2.90 (2.70 - 3.00)	2.65 (2.40 - 2.90)	0.011

Abbreviations: VAS, Visual Analog Scale; IQR, interquartile range.

^a^ Values are expressed as median (IQR).

^b^ Statistically signiﬁcant.

The VAS values at rest and during cough substantially decreased in group 2 than in group 1.

The results of the study showed that in group 2, the duration of stay in the recovery room was significantly reduced compared to group 1. Additionally, the number of patients requiring opioids and the total consumption of opioids were significantly lower in group 2 compared to group 1. Meanwhile, the study findings indicated that in group 2, the time to the first request for analgesia was significantly longer compared to group 1. Additionally, patient satisfaction levels were significantly higher in group 2 compared to group 1 (Table 4). 

**Table 4.** Postoperative Data Between the 2 Groups ^a, b^

Variables	Group 1 (N = 23)	Group 2 (N = 23)	P-Value
**Stay in the recovery room (h)**	3.62 ± 0.528	2.16 ± 0.493	< 0.001
**Patients required opioid, No. (%)**	16 (69.6)	3 (13)	< 0.001
**Time to request first analgesia (min)**	3.47 ± 1.58	6.33 ± 3.41	0.001
**Total opioid consumption (mg)**	12.37 ± 6.2	8.4 ± 4.62	0.029
**Patient satisfaction**	2.72 ± 0.781	4.35 ± 0.722	< 0.001
**Surgeon satisfaction**	2.65 ± 0.644	2.48 ± 0.639	0.374

^a^ Values are expressed as mean ± SD unless otherwise indicated.

^b^ Time to request first analgesia and patient satisfaction were substantially greater in group 2 than in group 1.

**Figure 8.**
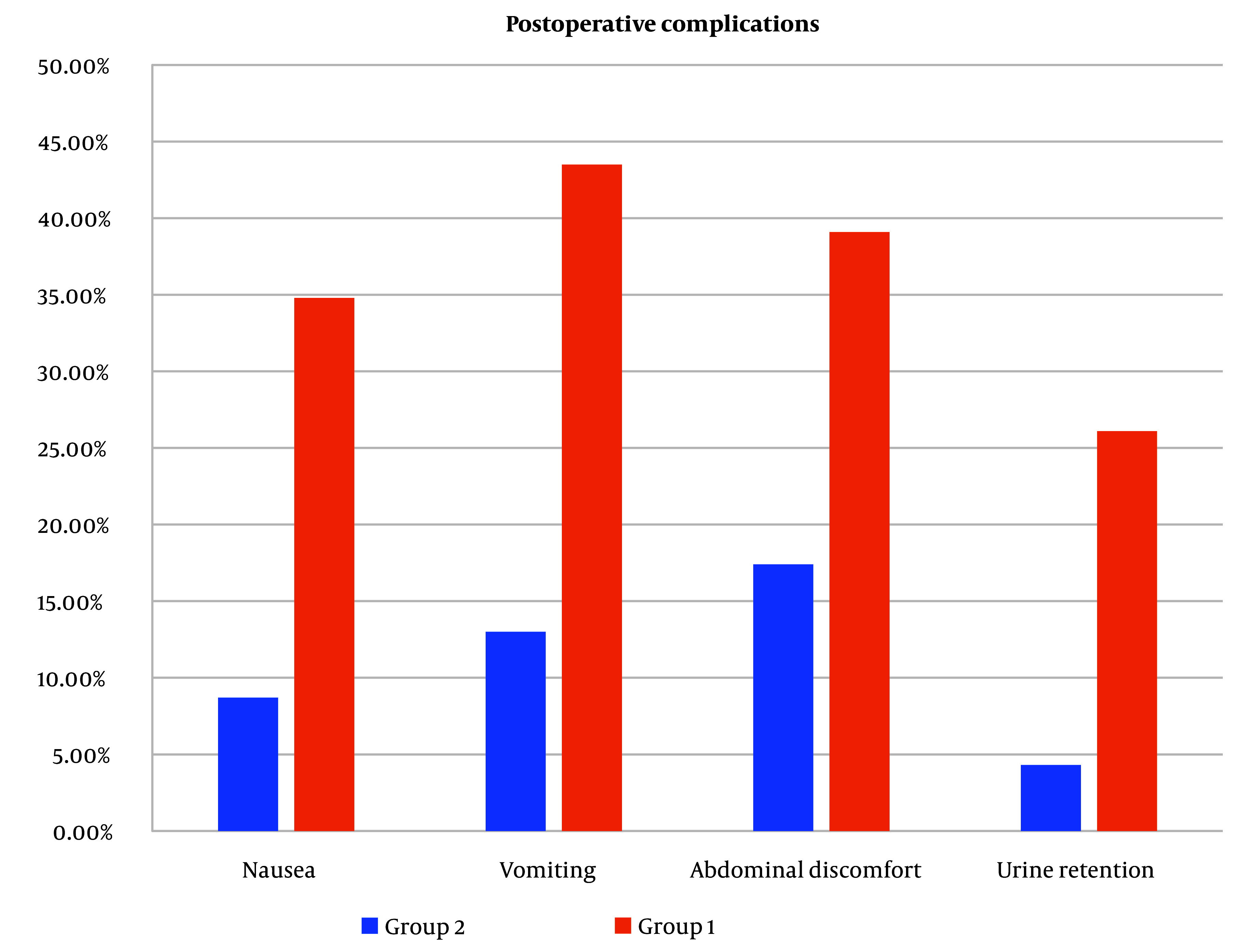
Postoperative complications between the 2 groups. Nausea, vomiting, and urine retention were substantially reduced in group 2 than in group 1. However, abdominal discomfort was lower among group 2 but without a statistically significant difference.

Nausea, vomiting, and urine retention were substantially reduced in group 2 than in group 1. However, abdominal discomfort was lower in group 2 but without a statistically significant difference (Figure 8). 

## 5. Discussion

The 2 main methods of regional anesthetic are epidural and spinal blocks. However, because of their intrinsic flaws, both have experienced highs and lows. Hypotension, post-dural-puncture headache, and unpredictability of block degree and duration are among the problems associated with SA. Similar to spinal blocks, epidural blocks may exhibit slower onsets of action, poor muscular relaxation, and insufficient analgesia, especially in terms of sacral segment-sparing efficacy (10).

The combined spinal-epidural technique is gaining popularity as a reliable, effective, and practical option among anesthesiologists. The procedure has been proven to be superior in cesarean delivery, labor analgesia, and orthopedic surgery of the abdomen and lower limbs (11).

Even when patients get fluid preload and ephedrine, spinal anesthetic alone may cause hypotension, particularly in older patients. A sequential mixed spinal epidural approach is used to lessen the likelihood of hypotension, using a spinal dosage of local anesthetic that is meant to be insufficient for operation. The epidural medication would prolong the block of cephalad. This approach does not postpone the onset of the block, but it does result in a sufficient amount of sensory block. This method was used in the profession of obstetric anesthesia, although orthopedic patients may also benefit from it (11).

In this investigation, we discovered a significant difference between the groups regarding intraoperative hypertension and tachycardia, in which HR and MAP measures were higher in group 1 than in group 2.

Our results are consistent with those of Eldaba and Amin (12), who observed a significant increase in HR and mean arterial blood pressure (MABP) in the GA group following intubation. This response may be attributed to the stress reaction triggered by laryngoscopy and intubation. Meanwhile, in the combined epidural spinal (CES) group, the hemodynamic parameters were essentially steady.

Furthermore, studies conducted by Abdallah et al. (13) and Nakano et al. (14) also support our findings, revealing that patients under GA exhibited higher HR and MABP compared to those undergoing regional blocks. In this research, we showed that VAS values were significantly lower in group 2 than in group 1.

Our results are consistent with the research conducted by Waters et al. (15), which aimed to assess surgeon and patient satisfaction with upper extremity blocks. The results of their study indicated excellent patient satisfaction, with a VAS score of 1.7 ± 2.3 on a 0- to 10-cm scale. Also, Ismail (16) found that the VAS score for patient satisfaction was also significantly reduced in the CSE group than in the general group (11.2 ± 7.304 vs 26.4 ± 22.94).

Similarly, Ellakany et al. (17) demonstrated that when compared to patients receiving GA, the median postoperative VAS at 4, 8, 12, and 24 h was considerably lower in the thoracic spinal-epidural group of patients.

The current study demonstrated that the duration of stay in the recovery room, the number of patients requiring opioids, and the total opioid consumption were significantly lower in group 2 compared to group 1. Meanwhile, the time to request the first analgesia and patient satisfaction were significantly higher in group 2 compared to group 1 in this study.

Our results were supported by Ismail (16), who found that compared to the CES group, the general group required significantly more additional analgesics and sedatives.

Moreover, Eldaba and Amin (12) reported that in the GA group, patients requested their first painkiller in a shorter amount of time compared to the spinal/epidural group; this difference was statistically significant (P < 0.05). Additionally, they found that patients in the CES group had higher levels of patient satisfaction compared to the GA group. It was observed that patients in the GA group received a higher amount of opioids for postoperative pain management, which was associated with an increased incidence of postoperative nausea and vomiting. This difference could be attributed to the fact that patients in the GA group experienced less postoperative vomiting and nausea due to the prolonged analgesia provided.

Also, Ellakany (17) demonstrated that patients in the CES group reported a median satisfaction score of 3.6, which was considerably higher than the 2.9 satisfaction score reported by patients in the GA group. The CES group's surgeon satisfaction score of 3 was substantially lower than the GA group's score of 4.1.

Additionally, Yayik et al. (18) revealed that a single dosage of tramadol was used on the second postsurgical day by 48% of the patients, and prolonged postsurgical analgesia lasting up to 24 h was linked to epidural anesthesia. In line with this study's findings, Tangpaitoon et al. (19) revealed that patients who received regional anesthesia reported higher patient satisfaction than those who received GA. Moreover, Bajwa et al. (20) revealed that patient satisfaction levels were higher with regional anesthetic, but surgeon satisfaction rates were similar in both groups.

In contrast to our research, Singhal et al. (21) revealed that in patients having a complete abdominal hysterectomy, there was no statistically significant difference between the general anesthetic group and the regional anesthetic group regarding surgeon and patient satisfaction rates.

This study had several limitations that should be acknowledged. First, we did not assess the amount of blood loss. Second, the follow-up period was relatively short. Lastly, the sample size was limited. Therefore, we need to conduct another research with a bigger sample size to confirm our findings.

### 5.1. Conclusions

Combined thoracic spinal/epidural block results in stable hemodynamics, longer postoperative analgesia with fewer side effects, and greater surgeon and patient satisfaction in patients undergoing abdominal operations and laparoscopic procedures.

## Data Availability

All authors choose to make data available upon request.

## Clinical Trial Registration Code:

NCT05587608.
